# Serum uric acid-to-HDL cholesterol ratio and stroke prevalence: NHANES 1999–2018 with external support from an imaging-confirmed hemorrhagic stroke dataset

**DOI:** 10.3389/fneur.2026.1798258

**Published:** 2026-06-26

**Authors:** Tiansheng Su, Yong Mo, Guangxiang Huang, Jiachao Lu, Shuling Tang, Qin Hu, Qianrong Huang, Fangzhou Guo, Ligen Mo, Jun Yan

**Affiliations:** 1 Department of Neurosurgery, Guangxi Medical University Cancer Hospital, Nanning, China; 2 Department of Neurosurgery, The First Affiliated Hospital of Guangxi Medical University, Nanning, China; 3 Department of Neurosurgery, Ren Ji Hospital, School of Medicine, Shanghai Jiao Tong University, Shanghai, China

**Keywords:** diabetes mellitus, NHANES, restricted cubic spline, stroke prevalence, UHR

## Abstract

Metabolic–inflammatory dysregulation contributes to cerebrovascular vulnerability, yet accessible composite indices for population-level stratification remain under active investigation. Using nationally representative data from U. S. adults in NHANES 1999–2018, we investigated the association between the uric acid to high-density lipoprotein cholesterol ratio (UHR) and prevalent stroke. Survey-weighted multivariable logistic regression and restricted cubic spline models were applied with comprehensive adjustment for demographic, metabolic, and socioeconomic factors. Higher UHR levels were independently associated with increased odds of prevalent stroke [1.02 (1.01–1.03)]. Individuals within the top UHR quartile exhibited roughly a 37% higher stroke prevalence compared with those in the lowest quartile [1.37 (1.17–1.61)]. Restricted Cubic Spline analyses demonstrated an approximately linear association between UHR and stroke prevalence (P for nonlinearity = 0.62). Cross-sectional discrimination assessed by survey-weighted ROC analysis yielded an AUC of 0.825, which should be interpreted as differentiation of prevalent stroke rather than prognostic prediction. Diabetes status significantly modified the association (P for interaction < 0.05), with a stronger relationship observed among non-diabetic individuals. To assess robustness across settings and phenotype definitions, we further evaluated UHR in an independent Chinese hospital-based cohort with imaging-confirmed hemorrhagic stroke, which showed directionally consistent associations. Collectively, these findings support UHR as a clinically accessible, hypothesis-generating marker capturing metabolic–inflammatory imbalance relevant to cerebrovascular pathology and motivate future longitudinal and mechanistic studies.

## 1 Introduction

Stroke continues to represent a major global contributor to the foremost causes of death and persistent disability. It remains a substantial contributor to the worldwide disease burden and exerts considerable economic pressure on healthcare systems, with estimated annual costs surpassing $89 billion (1, 2). Clinically, stroke is a neurological emergency marked by a sudden disruption of cerebral blood flow, resulting in either ischemic or hemorrhagic events (3). Ischemic stroke (IS) constitutes nearly 85% of all stroke occurrences and is primarily caused by arterial occlusion, whereas hemorrhagic stroke (HS) arises from the rupture of cerebral vessels (4). Regardless of stroke subtype, stroke poses a significant threat to patients’ health status and overall quality of life.

Although acute interventions including intravenous thrombolysis and endovascular thrombectomy have markedly improved outcomes in ischemic stroke, a substantial proportion of survivors continue to experience persistent neurological impairments, including motor, sensory, and cognitive deficits (5, 6). Accordingly, effective identification and regulation of modifiable risk factors may be essential to decreasing stroke occurrence and alleviating its chronic health burden (7). These considerations highlight the pressing importance of advancing research aimed at developing novel therapeutic strategies, early detection of reliable biomarkers, and effective preventive interventions to mitigate the worldwide burden of stroke.

Uric acid (UA) has recently attracted considerable interest as a key component involved in metabolic and vascular processes. Once regarded solely as an end-product of purine degradation, UA is now recognized as a notable pro-inflammatory molecule that activates multiple immune-inflammatory pathways (8). It promotes M1 macrophage polarization, enhances their migratory capacity, and concurrently suppresses phagocytic function (9). Elevated serum uric acid (SUA) concentrations have been associated with vascular endothelial impairment, heightened inflammatory activity, oxidative stress, and a heightened risk of cardiovascular events (10, 11). Analyses based on National Health and Nutrition Examination Survey (NHANES) further revealed that SUA levels were independently associated with all-cause and cardiovascular mortality, particularly among individuals with comorbid hypertension and diabetes (12). Mechanistic studies suggest that SUA may accumulate in atherosclerotic plaques, triggering chronic vascular inflammation, enhancing platelet adhesiveness, and promoting thrombus formation (13, 14).

In contrast, High-density lipoprotein (HDL), a lipoprotein particle composed mainly of apolipoprotein A-I (apoA-I), phospholipids, and cholesterol, is conventionally termed “good cholesterol” due to its cardioprotective properties (15). HDL exerts multiple atheroprotective effects, including facilitation of reverse cholesterol transport, transport of antioxidant enzymes like paraoxonase-1 (PON1) to counter lipid oxidation, inhibition of NF-κB-dependent inflammatory signaling, and enhancement of endothelial nitric oxide synthase (eNOS)-mediated vascular function (16). HDL-cholesterol (HDL-C), reflecting the cholesterol content of HDL particles, is routinely used as a biomarker in lipid profiling and serves as a fundamental biomarker for evaluating cardiovascular risk. Clinically, hyperuricemia is associated with conditions such as gout, nephropathy, and cardiovascular disease, while low HDL-C contributes to atherosclerosis and metabolic syndrome. The coexistence of these two metabolic abnormalities may synergistically exacerbate vascular injury and associated with higher odds of cerebrovascular events.

Given the opposing vascular implications of uric acid and HDL-C, recent research has identified the serum uric acid to high-density lipoprotein cholesterol ratio (UHR) as a potential integrated marker that may effectively reflect systemic metabolic imbalance and cardiometabolic risk (17). UHR has been recognized as a composite marker of the interplay between uric acid metabolism and lipid homeostasis, and has shown clinical utility in clinical settings for monitoring nonalcoholic fatty liver disease (NAFLD), metabolic syndrome, cardiovascular disease (CVD), diabetes, and chronic kidney disease (17–18). Despite prior analyses from NHANES having reported that higher UHR values may be linked to an increased likelihood of stroke, these studies were methodologically limited by relatively small sample sizes, the absence of complex sampling weights, the lack of multiple imputation for missing data (19) and the absence of independent external cohort validation, which collectively constrained the generalizability and robustness of their findings.

To address these limitations, the current analysis employed data from NHANES 1999–2018, encompassing 48,918 adults aged ≥20 years, and applied a survey-weighted modeling strategy to evaluate the nationwide relationship between UHR and stroke prevalence. Incomplete covariate information was addressed via multiple imputation by chained equations to mitigate bias introduced by missing data. The novelty of this study lies in several aspects. First, we used a large nationally representative sample with appropriate consideration of the complex NHANES survey design. Second, we evaluated UHR both as a continuous variable and by quartiles, and further used restricted cubic spline analysis to characterize the dose–response pattern. Third, we explored subgroup heterogeneity and identified diabetes status as a significant effect modifier of the UHR–stroke association. Fourth, we incorporated an independent hospital-based imaging-confirmed hemorrhagic stroke dataset as exploratory external supportive evidence to examine whether the observed association showed directional consistency in a clinically distinct stroke phenotype. Collectively, this study provides methodologically robust and hypothesis-generating epidemiological evidence regarding the relationship between UHR and stroke prevalence, and may inform future longitudinal, mechanistic, and translational investigations into metabolic dysregulation and cerebrovascular vulnerability.

## 2 Materials and methods

### 2.1 Population and study design

NHANES represents a continuous series of cross-sectional surveys (conducted biennially since 1999). Using a stratified, multistage probability sampling framework, it generates nationally representative information on the health and nutritional status of the US civilian, non-institutionalized population (20). In addition to laboratory tests, each participant received a household interview, physical examination, and other assessments. As a comprehensive national survey, it offers extensive data resources for examining the epidemiological characteristics of stroke within the American population. All survey procedures are reviewed and approved by the National Center for Health Statistics Research Ethics Review Board, and written informed consent is obtained from every participant. Comprehensive documentation on survey methodology, sampling procedures, and public data access is available on the NCHS website: www.cdc.gov/nchs/nhanes/.

### 2.2 Study population

**Figure 1:**
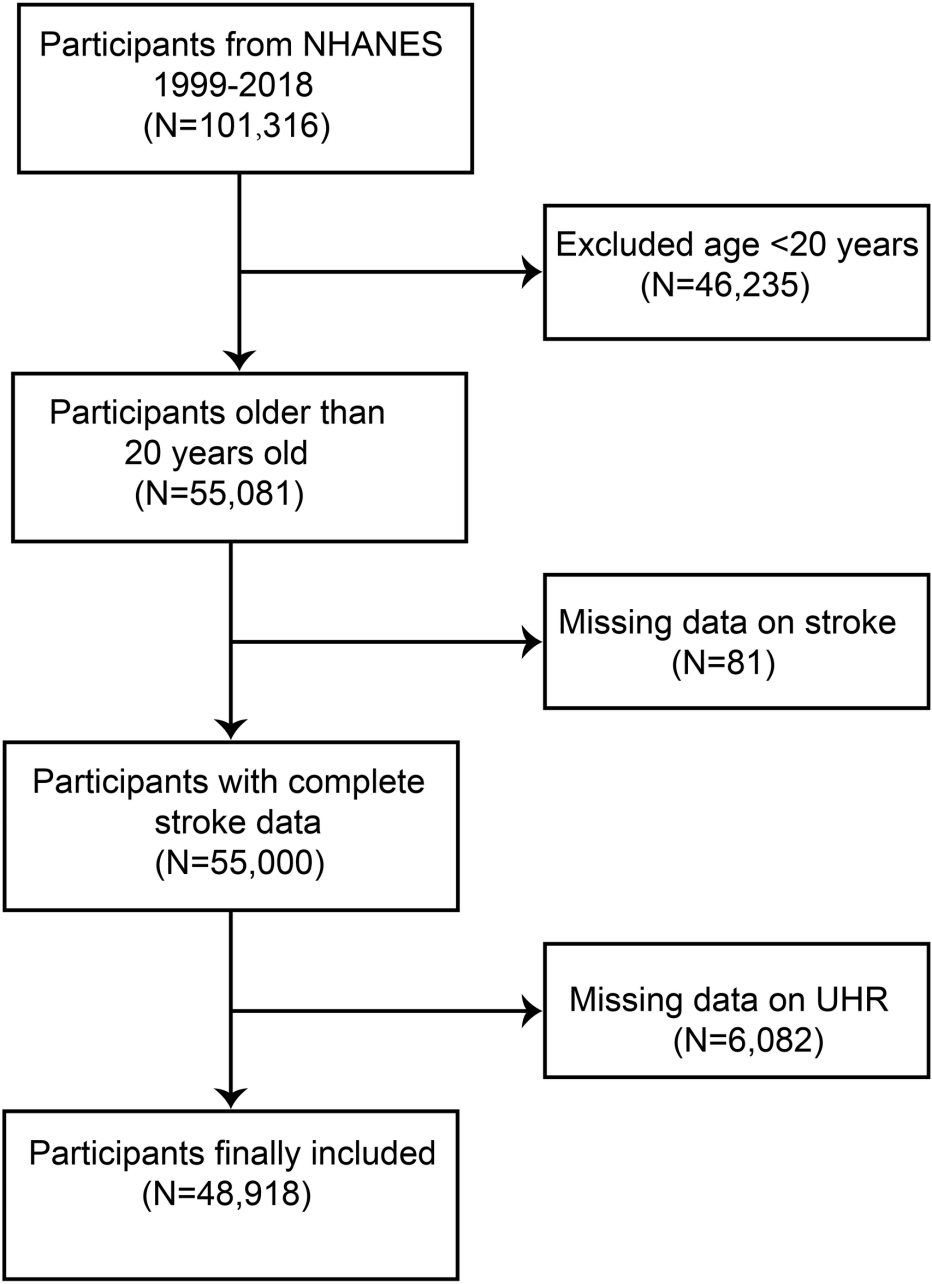
Flowchart of participant selection from NHANES 1999–2018. Inclusion: age ≥20 years. Exclusions: missing stroke status; missing SUA or HDL-C to compute UHR.

Based on the inclusion criteria of age ≥20 years and participation in any NHANES cycle between 1999 and 2018, a total of 55,081 adults were initially identified from 101,316 survey participants. Because this study was a secondary analysis of an existing nationally representative cross-sectional survey, no formal *a priori* sample size calculation was performed. Instead, all eligible participants meeting the predefined inclusion and exclusion criteria were included. The final analytic sample size was derived as follows: among the 101,316 participants available in NHANES 1999–2018, 46,235 individuals aged <20 years were excluded, leaving 55,081 adults eligible for further screening. We then excluded 6,163 participants with missing information on self-reported stroke status and/or missing serum uric acid or HDL-C data required for calculating UHR. Therefore, the final analytic sample included 48,918 participants (Figure 1). Individuals were excluded if they: (1) had missing information on self-reported stroke status, and/or (2) had missing SUA or HDL-C data required for calculating the UHR.

### 2.3 Assessment of UHR

Serum uric acid (SUA, mg/dL; laboratory code: LBXSUA) and HDL-cholesterol (HDL-C, mg/dL; laboratory code: LBDHDD) concentrations were retrieved from the NHANES laboratory dataset. The UHR was computed by dividing SUA by HDL-C and scaled by a factor of 100 for interpretability; therefore, all analyses and figures report UHR (21).

### 2.4 Diagnosis of stroke

Information on stroke status was obtained from the NHANES medical conditions questionnaire. A questionnaire regarding medical problems was utilized to evaluate the prevalence of stroke. Participants were classified as having a history of stroke if they answered “yes” to the question: “Has a doctor or other health professional ever told you that you had a stroke?” The validity of self-reported stroke in NHANES has been confirmed in prior studies (19). Stroke was identified as the primary outcome variable in the current research.

### 2.5 Covariates

In NHANES-based analyses, it is essential to adjust for potential confounders, including demographic factors such as age, gender, and race associated with both the exposure and the outcome, yet are not part of the causal pathway. Failure to adequately control for these confounders can introduce bias, leading to inaccurate estimations of the exposure-outcome relationship (22). Adjusting for covariates enhances the plausibility of genuine links by allowing researchers to more clearly isolate the independent effects of exposure variables on outcome variables. Furthermore, including appropriate covariates in statistical models enhances the explanatory power and discriminative performance accuracy of the models. Incorporating relevant covariates enables more precise model estimation and enhances the robustness and credibility of the findings. The covariates included in this study were age, sex, race/ethnicity, educational attainment, poverty-to-income ratio (PIR), smoking behavior, alcohol use, physical activity (PA), body mass index (BMI), hypertension, and diabetes mellitus. Detailed information on all variables is available in the NHANES public data repository.

### 2.6 RCS analysis

Restricted cubic spline analysis, a commonly used approach for flexibly modeling exposure–response relationships between continuous exposures and health outcomes in epidemiological studies, was performed to evaluate the dose–response association between UHR and prevalent stroke in the NHANES dataset (23). UHR was modeled as a continuous variable using three knots, with knot locations automatically determined according to the empirical distribution of UHR. The reference value was set at the median UHR based on the datadist object used in the rms framework. To account for the NHANES sampling structure at the weighting level, normalized examination weights were incorporated into the spline model. The overall association and non-linearity were assessed using Wald tests derived from the spline model. Odds ratios and 95% confidence intervals were plotted across the observed UHR range.

### 2.7 Subgroup analyses

Subgroup analyses were conducted to assess whether the association between UHR and prevalent stroke differed across prespecified participant characteristics, as recommended in epidemiological analyses evaluating potential population heterogeneity (24). The subgroup variables included sex, age group, race/ethnicity, education level, poverty-to-income ratio, drinking status, smoking status, diabetes, physical activity level, and hypertension. Within each subgroup, logistic regression models were adjusted for the same covariates as the fully adjusted model, except for the corresponding stratifying variable. Potential effect modification was assessed by including an interaction term between UHR and each subgroup variable. A two-sided P for interaction <0.05 was considered statistically significant.

### 2.8 Statistical analysis

We constructed survey design objects specifying examination weights (WTMEC2YR), strata, and primary sampling units for each 2-year cycle and concatenated cycles following NCHS guidance. Model adequacy was assessed using the Akaike and Bayesian information criteria (AIC and BIC), which were reported to two decimal places. Missing covariates were addressed with multiple imputation by chained equations (MICE), with the outcome included in the imputation model. UHR was evaluated both as a continuous measure (per 1-unit increase in UHR), with UHR defined as SUA/HDL-C × 100; thus, each 1-unit increment represents a 0.01 increase in the SUA/HDL-C ratio. For clinical interpretation, if HDL-C is held constant at 50 mg/dL, a 1-unit increase in UHR would correspond approximately to a 0.5 mg/dL increase in serum uric acid. Conversely, if serum uric acid is held constant at 5 mg/dL, an increase in UHR from 10 to 11 would correspond approximately to a decrease in HDL-C from 50.0 mg/dL to 45.5 mg/dL. These examples are illustrative because UHR is determined jointly by both serum uric acid and HDL-C. Survey-weighted logistic regression models were fitted to explore the relationship between UHR and stroke across three progressively adjusted model sets. Discriminatory ability was quantified using receiver operating characteristic (ROC) curves with 95% confidence intervals. A two-sided *p* < 0.05 was regarded as statistically significant. All analyses were conducted using R 4.5.0 and EmpowerStats 2.0.

### 2.9 Exploratory hospital-based hemorrhagic stroke analysis

Patients were consecutively identified from the Department of Neurosurgery, The First Affiliated Hospital of Guangxi Medical University between January and September 2025. Patients with trauma-related hemorrhage, intracranial infections, neoplastic diseases, postoperative conditions, or other non-target diagnoses were excluded. The comparison group consisted of non-hemorrhagic and non-ischemic neurosurgical inpatients in whom intracranial hemorrhage was excluded by admission CT or MRI. This hospital-based analysis represents a comparison between imaging-confirmed hemorrhagic stroke patients and non-hemorrhagic, non-ischemic neurosurgical inpatients. Cases with missing serum uric acid or HDL-C data required for UHR calculation were further excluded. The final analytical cohort was established after the stepwise screening process, as illustrated in Supplementary Figure S1, with a total of 201 patients included. Participants were categorized into UHR quartiles using cohort-specific cut-off values, which are reported in Supplementary Table S1. Logistic regression models were applied to examine the association between UHR and hemorrhagic stroke. Consistent with the primary NHANES analysis and to reduce the risk of overfitting given the limited sample size, UHR was analyzed both as a continuous variable and by quartiles, with adjustment for age, sex, body mass index, hypertension, and diabetes mellitus. The external validation analysis was conducted only to examine directional and pattern-level consistency of the association between UHR and hemorrhagic cerebrovascular pathology in a clinically distinct imaging-confirmed phenotype. It was not intended for population-level prevalence estimation, stroke subtype comparison, predictive modeling, or causal inference.

## 3 Results

### 3.1 Baseline characteristics

The research involved 48,918 individuals with an average age of 46.93 ± 16.90 years, and 48.22% were men. Overall, 1,840 individuals (3.76%) reported a history of stroke. Table 1 provides a summary of the participants’ baseline characteristics. The mean UHR for the entire cohort was 13.82 ± 2.63, with the following interquartile cut-offs: quartile 1 (<7.54), quartile 2 (7.54–10.44), quartile 3 (10.44–14.20), and quartile 4 (≥14.20). Participants in the higher UHR quartiles tended to be slightly older (Q4: 46.99 ± 16.80 vs. Q1: 46.51 ± 16.68), predominantly male (79.86% in Q4 vs. 13.73% in Q1), lower educational attainment (53.39% with education beyond high school in Q4 vs. 66.12% in Q1), and were more likely to be current smokers (51.97% in Q4 vs. 39.82% in Q1). Additionally, participants in the higher UHR quartiles showed higher prevalences of diabetes (18.42% in Q4 vs. 6.24% in Q1) and hypertension (46.88% in Q4 vs. 28.08% in Q1), as well as a higher prevalence of stroke. Elevated UHR values were associated with higher body mass index, waist circumference, LDL-C, triglycerides, and C-reactive protein levels, accompanied by markedly lower HDL-C concentrations (all *p* < 0.001). In contrast, the proportion of participants with high PIR decreased across increasing UHR quartiles (46.53% in Q1 vs. 39.84% in Q4). Further analysis of the different demographic characteristics in Table 2 showed that factors such as advanced age, female gender, limited education, poverty, smoking status, hypertension, and diabetes were notably connected to an increased occurrence of stroke. Furthermore, CRP (0.59 ± 1.25 mg/L vs. 0.38 ± 0.87 mg/L), FPG (117.03 ± 42.89 mg/dL vs. 104.57 ± 30.20 mg/dL), HbA1c (6.05 ± 1.27% vs. 5.56 ± 0.90%), and UHR (14.58 ± 2.77 vs. 13.74 ± 2.61) were significantly elevated in stroke patients compared to non-stroke individuals (all *p* < 0.0001), while triglyceride (TG) levels did not differ significantly (*p* = 0.7093).

**Table 1:** Baseline characteristics according to UHR quartiles.

Characteristics	Q1 (<7.54)	Q2 (7.54–10.44)	Q3 (10.44–14.20)	Q4 (≥14.20)	*p*-value
Age (years)	46.51 ± 16.68	46.91 ± 17.24	47.33 ± 16.89	46.99 ± 16.80	0.0018
Gender					<0.001
Male	1,678 (13.73)	4,690 (38.31)	7,585 (61.88)	9,767 (79.86)	
Female	10,543 (86.27)	7,549 (61.69)	4,648 (38.12)	2,458 (20.14)	
Race					<0.001
Mexican American	854 (6.98)	1,040 (8.49)	1,069 (8.74)	1,046 (8.54)	
Other Hispanic	637 (5.21)	724 (5.91)	703 (5.74)	712 (5.82)	
Non-Hispanic White	8,494 (69.50)	8,197 (66.98)	8,360 (68.31)	8,506 (69.50)	
Non-Hispanic Black	1,420 (11.62)	1,462 (11.95)	1,271 (10.39)	1,074 (8.78)	
Other Race	819 (6.70)	815 (6.67)	833 (6.81)	900 (7.36)	
Education					<0.001
Less than high school	1,679 (13.74)	2,193 (17.90)	2,198 (17.97)	2,389 (19.52)	
High school	2,463 (20.14)	2,918 (23.85)	3,038 (24.80)	3,312 (27.09)	
More than high school	8,079 (66.12)	7,128 (58.25)	7,007 (57.22)	6,524 (53.39)	
Poverty-income ratio					<0.001
Low	2,322 (18.98)	2,791 (22.79)	2,688 (21.98)	2,759 (22.56)	
Moderate	4,220 (34.49)	4,401 (35.98)	4,460 (36.48)	4,594 (37.60)	
High	5,679 (46.53)	5,043 (41.24)	5,084 (41.55)	4,872 (39.84)	
Drinking status					<0.001
Never	2,428 (19.85)	2,494 (20.38)	2,385 (19.42)	2,390 (19.56)	
Moderate	4,411 (36.07)	4,700 (38.37)	4,998 (40.84)	5,037 (41.18)	
Heavy	5,382 (44.08)	5,045 (41.25)	4,850 (39.74)	4,798 (39.26)	
Smoking status					<0.001
No	7,358 (60.18)	6,871 (56.18)	6,196 (50.64)	5,871 (48.03)	
Yes	4,863 (39.82)	5,368 (43.82)	6,037 (49.36)	6,354 (51.97)	
Physical activity level					<0.001
Low	3,113 (25.46)	3,107 (25.36)	3,248 (26.55)	3,439 (28.13)	
Moderate	5,036 (41.20)	4,818 (39.36)	4,559 (37.26)	4,347 (35.52)	
High	4,072 (33.34)	4,314 (35.28)	4,426 (36.19)	4,448 (36.35)	
Hypertension					<0.001
No	8,791 (71.92)	8,014 (65.47)	7,321 (59.82)	6,493 (53.12)	
Yes	3,430 (28.08)	4,225 (34.53)	4,912 (40.18)	5,732 (46.88)	
Diabetes					<0.001
No	11,456 (93.76)	10,957 (89.59)	10,534 (86.09)	9,973 (81.58)	
Yes	765 (6.24)	1,282 (10.41)	1,699 (13.91)	2,252 (18.42)	
Body mass index (kg/m^2^)	25.50 ± 5.40	28.16 ± 6.33	29.74 ± 6.68	31.78 ± 6.76	<0.001
C-reactive protein (mg/L)	0.32 ± 0.80	0.37 ± 0.90	0.40 ± 0.83	0.47 ± 0.99	<0.001
Waist (cm)	88.51 ± 13.29	96.07 ± 14.66	101.60 ± 15.18	108.12 ± 15.55	<0.001
Fasting plasma glucose (mg/dL)	99.47 ± 25.73	103.30 ± 29.24	106.91 ± 31.96	110.11 ± 34.22	<0.001
Glycated hemoglobin A1c (%)	5.40 ± 0.80	5.54 ± 0.90	5.63 ± 0.94	5.73 ± 1.00	<0.001
HDL-C (mg/dL)	70.86 ± 15.73	56.09 ± 10.14	47.43 ± 7.88	37.94 ± 7.12	<0.001
LDL-C (mg/dL)	105.10 ± 38.73	114.66 ± 41.58	120.88 ± 42.88	126.40 ± 45.82	<0.001
Total cholesterol (mg/dL)	199.55 ± 38.52	195.30 ± 40.96	195.07 ± 42.80	194.95 ± 44.88	<0.001
Triglycerides (mg/dL)	120.39 ± 85.19	123.95 ± 99.43	133.53 ± 107.22	152.44 ± 153.67	<0.001

Data are mean ± SD for continuous variables and n (column %) for categorical variables. Percentages sum to 100% within columns unless stated. HDL-C and LDL-C, high- and low-density lipoprotein cholesterol; UHR = (SUA [mg/dL] /HDL-C [mg/dL]) × 100.

**Table 2:** Baseline demographic and socioeconomic features of participants stratified by stroke status.

Characteristics	Total (*N* = 48,918)	Non-stroke (*N* = 47,078)	Stroke (*N* = 1840)	*p-*value
Age (years)	46.93 ± 16.90	46.43 ± 16.70	64.70 ± 14.37	<0.001
Gender				<0.001
Male	23,588 (48.22%)	22,776 (48.38%)	783 (42.54%)	
Female	25,330 (51.78%)	24,302 (51.62%)	1,057 (57.46%)	
Race				<0.001
Mexican American	4,001 (8.18%)	3,903 (8.29%)	79 (4.28%)	
Other Hispanic	2,774 (5.67%)	2,698 (5.73%)	59 (3.21%)	
Non-Hispanic White	33,553 (68.59%)	32,248 (68.50%)	1,315 (71.46%)	
Non-Hispanic Black	5,229 (10.69%)	4,986 (10.59%)	258 (14.01%)	
Other Race	3,366 (6.88%)	3,239 (6.88%)	130 (7.04%)	
Education				<0.001
Less than high school	8,438 (17.25%)	7,966 (16.92%)	534 (29.03%)	
High school grade	11,711 (23.94%)	11,200 (23.79%)	538 (29.26%)	
More than high school	28,769 (58.81%)	27,913 (59.29%)	767 (41.71%)	
Poverty-income ratio				<0.001
Low	10,546 (21.55%)	9,992 (21.24%)	601 (32.68%)	
Moderate	17,668 (36.12%)	16,915 (35.91%)	806 (43.76%)	
High	20,704 (42.33%)	20,171 (42.86%)	433 (23.57%)	
Drinking status				<0.001
Never	9,693 (19.80%)	9,097 (19.33%)	670 (36.36%)	
Moderate	19,136 (39.09%)	18,387 (39.08%)	729 (39.72%)	
Heavy	20,089 (41.11%)	19,592 (41.59%)	441 (23.92%)	
Smoking status				<0.001
No	26,325 (53.79%)	25,473 (54.13%)	769 (41.81%)	
Yes	22,593 (46.21%)	21,605 (45.87%)	1,071 (58.19%)	
Physical activity level				<0.001
Low	12,895 (26.37%)	12,110 (25.73%)	906 (49.23%)	
Moderate	18,761 (38.35%)	18,102 (38.48%)	625 (33.94%)	
High	17,262 (35.27%)	16,866 (35.80%)	310 (16.83%)	
Hypertension				<0.001
No	30,642 (62.64%)	30,045 (63.82%)	384 (20.86%)	
Yes	18,276 (37.36%)	17,033 (36.18%)	1,456 (79.14%)	
Diabetes				<0.001
No	42,950 (87.80%)	41,631 (88.43%)	1,201 (65.27%)	
Yes	5,968 (12.20%)	5,447 (11.57%)	639 (34.73%)	
Body mass index (kg/m2)	28.77 ± 6.72	28.74 ± 6.71	29.87 ± 6.87	<0.001
C-reactive protein (mg/L)	0.39 ± 0.89	0.38 ± 0.87	0.59 ± 1.25	<0.001
Waist (cm)	98.51 ± 16.42	98.36 ± 16.36	103.72 ± 16.16	<0.001
Fasting plasma glucose (mg/dL)	104.91 ± 30.85	104.57 ± 30.20	117.03 ± 42.89	<0.001
Glycated hemoglobin A1c (%)	5.57 ± 0.92	5.56 ± 0.90	6.05 ± 1.27	<0.001
HDL-C (mg/dL)	53.20 ± 16.29	53.25 ± 16.27	51.72 ± 16.64	0.0007
LDL-C (mg/dL)	116.68 ± 43.09	116.83 ± 42.95	111.48 ± 46.07	<0.001
Total cholesterol (mg/dL)	196.25 ± 41.93	196.42 ± 41.74	190.01 ± 46.19	<0.001
Triglycerides (mg/dL)	132.52 ± 114.69	132.48 ± 115.13	133.67 ± 102.79	0.7093
UHR	13.82 ± 2.63	13.74 ± 2.61	14.58 ± 2.77	<0.001

Data are mean ± SD for continuous variables and n (column %) for categorical variables. Percentages sum to 100% within columns unless stated. HDL-C and LDL-C, high- and low-density lipoprotein cholesterol; UHR = (SUA [mg/dL] / HDL-C [mg/dL]) × 100.

### 3.2 Association between UHR and stroke

Table 3 illustrates the connection between UHR and the odds of prevalent stroke. In model 1, which is unadjusted, a significant positive correlation was identified between UHR and stroke.

**Table 3:** The association of UHR with stroke.

Exposure	OR (95%CI), *p*-value		
	Model 1^a^	Model 2^b^	Model 3^c^
UHR	1.05 (1.04, 1.05) < 0.001	1.04 (1.04, 1.05) < 0.001	1.02 (1.01, 1.03) < 0.001
UHR quartile			
Q1	1.0	1.0	1.0
Q2	1.39 (1.20, 1.61) < 0.001	1.28 (1.10, 1.49) 0.0017	1.16 (0.99, 1.35) 0.0643
Q3	1.52 (1.32, 1.76) < 0.001	1.41 (1.21, 1.64) < 0.001	1.16 (0.99, 1.36) 0.0631
Q4	2.03 (1.77, 2.33) < 0.001	1.90 (1.63, 2.21) < 0.001	1.37 (1.17, 1.61) 0.0001
AIC	12262.05	10608.63	10151.58
BIC	12281.19	10661.95	10253.77
Pseudo R^2^	0.005	0.140	0.178
AUC	0.576	0.794	0.825

a No-adjusted model: unadjusted.

b Minimally adjusted model: + age, sex, race/ethnicity, education.

c Fully adjusted model: Model 2 + PIR, BMI, smoking, drinking, physical activity, hypertension, diabetes.

**Figure 2:**
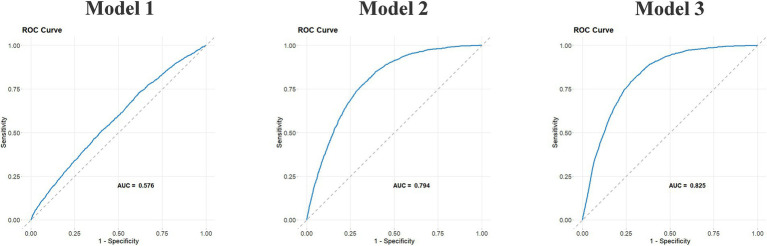
Survey-weighted ROC curves. Model 1: adjusted for none. Model 2: adjusted for age, sex, race, and education. Model 3: additionally adjusted for PIR, BMI, smoking, drinking, physical activity, hypertension, and diabetes based on Model 2. AUC, area under the curve.

[1.05 (1.04–1.05)]. After partial adjustment for age, gender, race, and education (Model 2), the association was attenuated but remained statistically significant: each 1-unit increase in UHR, corresponding to a 0.01 increase in the raw SUA/HDL-C ratio, was associated with a 2% higher odds of prevalent stroke [1.04 (1.04–1.05)]. In the fully adjusted model (Model 3), the association was attenuated but remained statistically significant, with a 2% increase in stroke prevalence for each unit rise in UHR [1.02 (1.01–1.03)]. When categorized into quartiles, individuals in Q4 exhibited higher odds of having stroke than those in Q1, with an estimated OR of 1.37 [1.37 (1.17–1.61)], while Q2 and Q3 did not show statistically significant differences. Model 3 demonstrated the best fit, evidenced by the lowest AIC and BIC values and the highest pseudoR^2^ (0.178), indicating relatively good explanatory power. Survey-weighted ROC analysis showed an AUC of 0.825 (Model 3), indicating acceptable discriminative performance for prevalent stroke (Figure 2).

**Figure 3:**
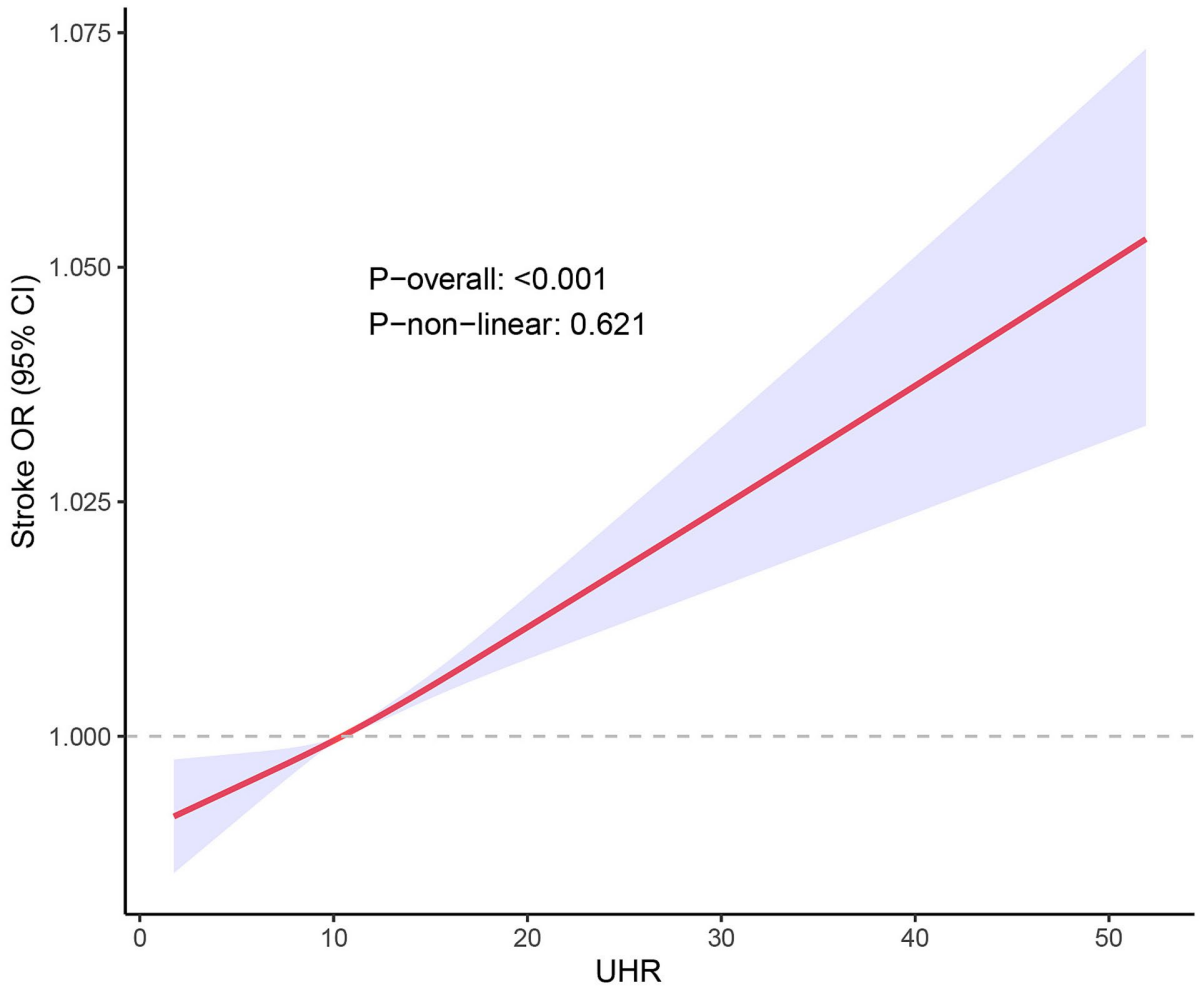
Restricted cubic spline (RCS) curve showing the association between UHR and stroke prevalence. The X-axis (exposure) represents UHR values; the Y-axis (outcome) represents odds ratios with 95% confidence intervals.

The RCS analysis yielded a significant overall association (P-overall < 0.001), while the nonlinearity test was nonsignificant (P for nonlinearity = 0.62), indicating that the exposure–outcome relationship was adequately described by a linear trend between UHR and the prevalence of stroke. Figure 3 illustrates a significant positive association between the UHR index and the odds ratio (OR) for stroke prevalence, with an overall *p*-value of less than 0.001. An elevation in the UHR index was associated with higher odds in the OR for stroke, as indicated by the OR curve increased monotonically; the shaded band denotes 95% CIs; P-overall<0.001; P for nonlinearity = 0.621 indicates approximate linearity. All model components had variance inflation factors (VIFs) between 1.09 and 1.57, which are well below the conventional cutoff of 5, indicating the absence of multicollinearity issues among the covariates.

### 3.3 Subgroup analysis

**Figure 4:**
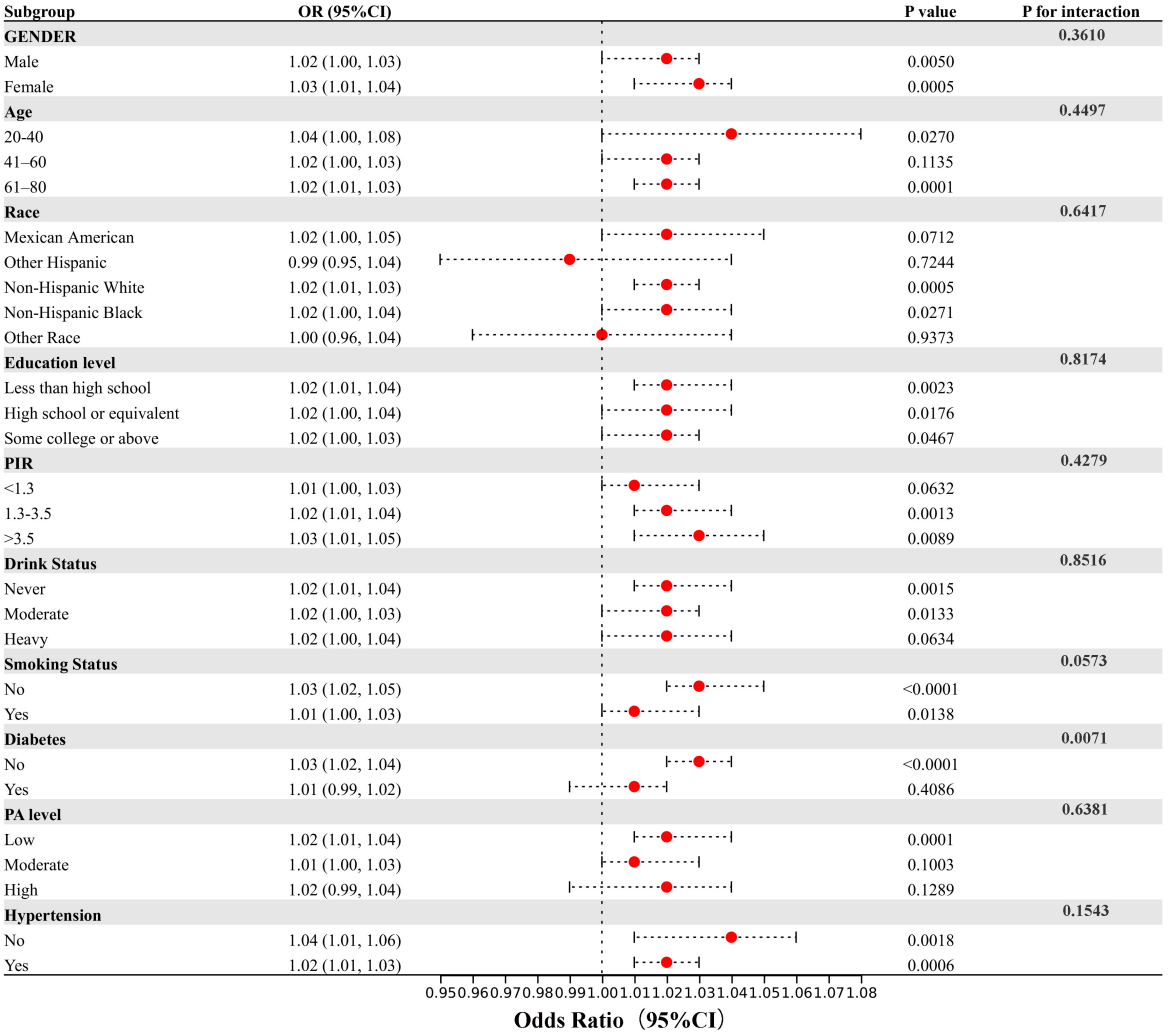
Subgroup analysis of the association between UHR and stroke (Model 3).

The interaction effect between UHR and each covariate on stroke prevalence was investigated through subgroup analysis. Forest plots (Figure 4) visualize the OR and 95% confidence interval (CI). In all subgroup analyses, the interaction test for the diabetes group was the only one with a *p*-value (*p* = 0.0071) below 0.05, suggesting that diabetes significantly influenced the association between UHR and stroke prevalence. The OR for UHR was notably higher in non-diabetic individuals compared to diabetic individuals, suggesting a more pronounced association between UHR and stroke prevalence among those without diabetes.

### 3.4 Exploratory external analysis in a Chinese imaging-confirmed hemorrhagic stroke dataset

Baseline characteristics of the exploratory hospital-based dataset stratified by UHR quartiles are presented in Supplementary Table S1. Across increasing UHR categories, participants exhibited higher body mass index, lower HDL-C concentrations, and higher serum uric acid and creatinine levels (all P for trend < 0.05), reflecting a metabolic profile broadly consistent with that observed in the primary NHANES cohort. When stratified by hemorrhagic stroke status, individuals with hemorrhagic stroke showed a higher prevalence of hypertension and modestly higher mean UHR levels compared with those without hemorrhagic stroke (Supplementary Table S2). In multivariable logistic regression analyses, UHR demonstrated a directionally positive association with hemorrhagic stroke when modeled as a continuous variable, although the association did not reach statistical significance after adjustment, likely reflecting the limited sample size of the external cohort (Supplementary Table S3). Consistently, when UHR was analyzed in quartiles, participants in the higher UHR categories exhibited numerically elevated odds of hemorrhagic stroke compared with those in the lowest quartile, with the strongest trend observed in the third quartile, albeit without statistical significance. Restricted cubic spline analysis further suggested a non-linear pattern, characterized by an initial increase in hemorrhagic stroke odds followed by attenuation at higher UHR levels (P for non-linearity = 0.094; Supplementary Figure S2). Taken together, although statistical power was limited, the external validation cohort demonstrated directional and pattern-level consistency with the primary NHANES analysis, suggesting potential directional consistency of the observed association between UHR and hemorrhagic stroke risk.

## 4 Discussion

In this nationally representative analysis of U. S. adults, elevated UHR was consistently associated with a higher prevalence of overall self-reported stroke after comprehensive multivariable adjustment. This association was robust across most demographic subgroups and remained stable in sensitivity analyses, supporting the epidemiological relevance of UHR as an integrated metabolic indicator. Notably, diabetes status emerged as a significant effect modifier, with a substantially stronger association observed among non-diabetic individuals. The external hospital-based analysis further provided exploratory directional support in an imaging-confirmed hemorrhagic stroke phenotype, although this finding should be interpreted separately from the NHANES-based overall stroke analysis.

Our findings are broadly consistent with previous studies reporting a positive linear association between UHR and stroke prevalence (e.g., Jiang et al. and Zhu et al.) (25, 26). However, earlier analyses were limited by relatively smaller sample sizes, incomplete consideration of complex survey design, or suboptimal handling of missing data. By leveraging the largest nationally representative US cohort to date (*n* = 48,918) and applying survey-weighted modeling with multiple imputation, the present study provides more stable and methodologically robust epidemiological evidence supporting this association. Importantly, the observed discriminative performance should be interpreted as cross-sectional differentiation rather than prognostic prediction or causal inference.

The external hospital-based analysis provided exploratory support using a clinically distinct imaging-confirmed hemorrhagic stroke phenotype. Although this external cohort differed substantially from the NHANES population in ethnicity, healthcare setting, and disease spectrum, elevated UHR demonstrated a consistent directional association with hemorrhagic cerebrovascular pathology. The external validation analysis was not designed to replicate effect sizes, establish predictive performance, or infer causality. Instead, it aimed to assess whether the epidemiological signal observed in a nationally representative population could be detected in a clinically distinct cohort with a well-defined hemorrhagic stroke phenotype. The observed trends across UHR quartiles and the pattern suggested by spline analysis provide supportive, hypothesis-generating evidence that UHR captures an underlying metabolic–vascular imbalance relevant to cerebrovascular vulnerability. Taken together, the convergence of population-based evidence from NHANES and hospital-based observations strengthens the biological plausibility of the association between UHR and cerebrovascular pathology while underscoring the exploratory nature of the present findings.

As an integrated biomarker, UHR may represent a promising indicator for evaluating the cumulative impact of systemic inflammation and metabolic dysregulation on cerebrovascular pathology, as it reflects both uric acid metabolism abnormalities and lipid profile imbalance (27, 28). Elevated uric acid contributes to oxidative stress, endothelial inflammation, and impaired nitric oxide signaling (29, 30), whereas HDL particles exert vasculoprotective effects through cholesterol efflux, anti-inflammatory activity, and preservation of endothelial function (31, 32). Consequently, an elevated UHR may reflect a systemic metabolic–inflammatory milieu characterized by excessive oxidative burden and insufficient endothelial protection, providing a biologically plausible link to cerebrovascular pathology.

**Figure 5:**
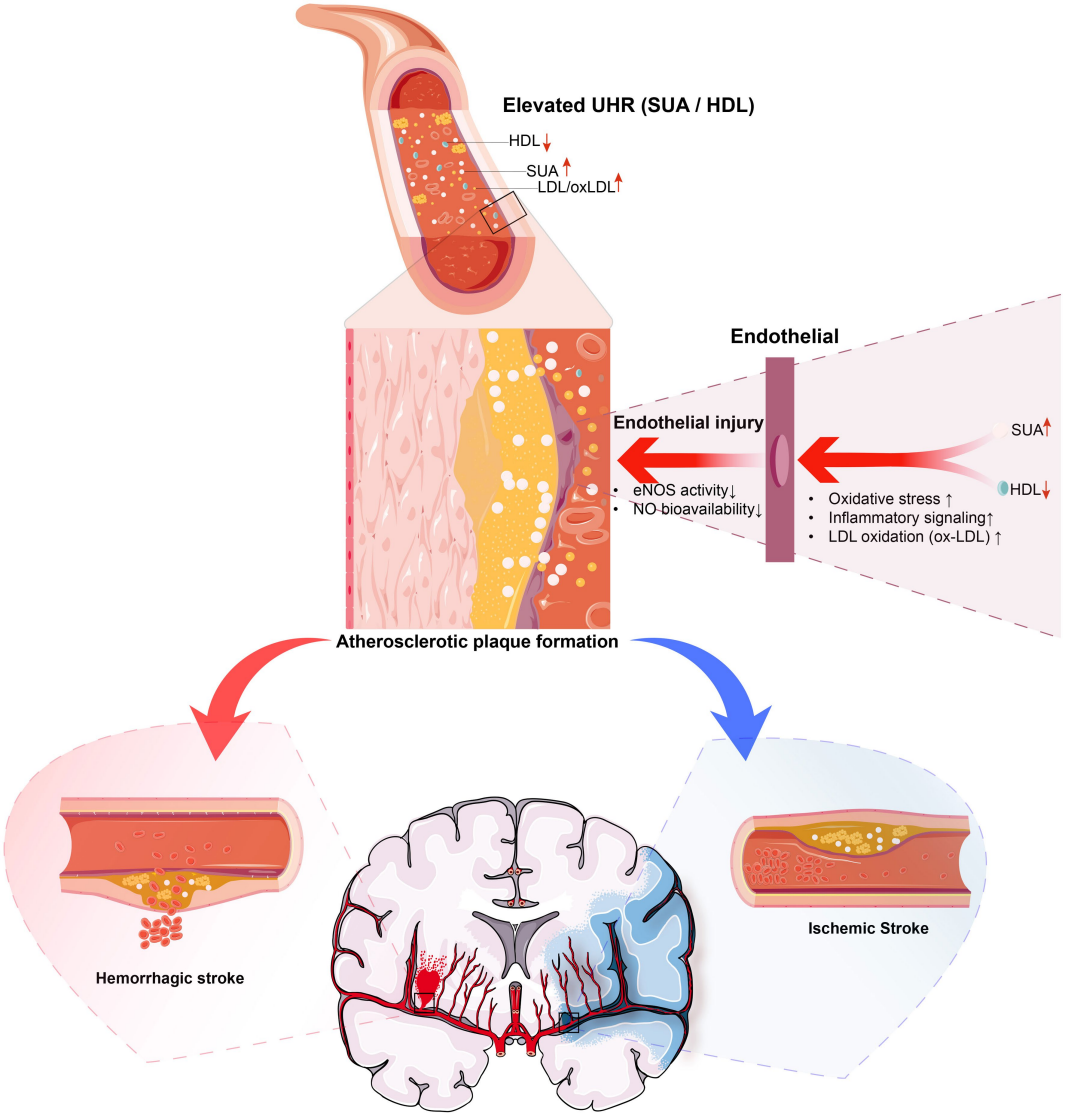
Proposed hypothesis-generating molecular framework linking elevated UHR to cerebrovascular vulnerability. Elevated UHR, reflecting increased serum uric acid and reduced HDL-cholesterol, may be associated with oxidative stress, inflammatory signaling, and endothelial dysfunction. These processes may facilitate atherosclerotic plaque formation and vascular instability, thereby potentially increasing susceptibility to ischemic and hemorrhagic stroke. This schematic illustrates biologically plausible pathways and does not represent direct mechanistic evidence derived from the present study.

Based on existing experimental and translational evidence, we propose a hypothesis-generating molecular framework to contextualize the observed epidemiological association between elevated UHR and cerebrovascular pathology (Figure 5). Elevated uric acid may promote oxidative stress, endothelial inflammation, impaired nitric oxide signaling, and vascular dysfunction, whereas reduced HDL-C may indicate weakened anti-inflammatory, antioxidative, and endothelial-protective capacity. Together, these changes may reflect a systemic metabolic-inflammatory milieu characterized by excessive oxidative burden and insufficient vascular protection (33). For ischemic stroke, such processes may facilitate endothelial dysfunction, atherosclerotic plaque formation, vascular inflammation, and thrombotic susceptibility. For hemorrhagic stroke, the same metabolic-inflammatory imbalance may contribute to small-vessel injury, blood–brain barrier disruption, vascular fragility, and hypertension-related cerebrovascular remodeling, thereby increasing susceptibility to vessel rupture. These mechanisms provide biological plausibility for the association of UHR with overall stroke prevalence in NHANES and for the directionally positive association observed in the exploratory hospital-based imaging-confirmed hemorrhagic stroke analysis. However, these mechanistic links remain speculative and were not directly tested in the present study; therefore, the hemorrhagic stroke findings should be interpreted as supportive and hypothesis-generating rather than definitive evidence of causality.

A key novel finding of this study is the identification of diabetes status as a significant effect modifier of the association between UHR and stroke prevalence, with a markedly stronger relationship observed among non-diabetic individuals. Several molecular mechanisms may plausibly account for this differential effect. In diabetic states, chronic hyperglycemia, insulin resistance, and sustained systemic inflammation may induce a partial “ceiling effect,” in which vascular injury and oxidative stress are already substantial, thereby attenuating the incremental contribution of UHR to stroke susceptibility (34). In addition, diabetes-related metabolic and inflammatory disturbances may alter lipoprotein structure and function, thereby attenuating the reliability of conventional lipid measures. In diabetic states, chronic hyperglycemia and oxidative stress have been shown to modify lipoproteins and complicate the interpretation of HDL-C–based indices, as HDL-C concentration alone may not fully reflect HDL functionality under conditions of metabolic stress (35). Although certain HDL subfractions, particularly HDL3, have been shown to possess superior antioxidative and endothelial-protective properties under physiological conditions, diabetes-associated glycation and oxidative modification of HDL particles substantially impair HDL functionality (36, 37). Experimental and clinical studies have demonstrated that such modifications can markedly reduce cholesterol efflux capacity—by approximately 30–40%—thereby attenuating the vasoprotective effects of HDL in diabetic individuals (38, 39). In contrast, among non-diabetic individuals, elevated UHR may reflect earlier metabolic–inflammatory disturbances and incipient endothelial dysfunction, thereby serving as a more sensitive indicator of cerebrovascular vulnerability. These findings suggest that UHR may be particularly informative as a population-level indicator of metabolic vulnerability in metabolically less advanced populations, whereas its discriminative value in diabetic individuals may be attenuated by pervasive metabolic dysregulation and the modifying effects of long-term pharmacologic interventions (40).

Several limitations warrant consideration. First, the cross-sectional design precludes causal inference, and the observed associations should not be interpreted as evidence that elevated UHR causes stroke. Second, residual confounding from unmeasured factors such as diet, genetic predisposition, or inflammatory mediators cannot be excluded. Third, stroke status in NHANES was self-reported and did not distinguish between ischemic and hemorrhagic subtypes. Although self-reported stroke has been widely used and has reasonable validity in epidemiological studies, it may still introduce recall or misclassification bias. Participants with undiagnosed stroke, mild stroke, remote stroke events, poor recall, or misunderstanding of prior medical diagnoses may have been misclassified. If such misclassification was largely non-differential with respect to UHR, the observed association would likely be biased toward the null, potentially leading to underestimation of the true association. However, differential misclassification cannot be completely excluded, because individuals with more comorbidities or more frequent healthcare contact may be more likely to report a physician diagnosis of stroke. Therefore, the primary NHANES findings should be interpreted as associations with overall self-reported stroke prevalence rather than ischemic or hemorrhagic stroke specifically. Finally, the external validation cohort was hospital-based, and control participants were derived from a neurosurgical inpatient population rather than the general community, which may limit generalizability and introduce selection bias. Despite these limitations, the present study provides population-level and externally supported evidence suggesting that UHR is a practical, hypothesis-generating biomarker reflecting systemic metabolic and inflammatory imbalance with relevance to cerebrovascular pathology. These findings may inform future longitudinal and mechanistic studies aimed at elucidating how metabolic dysregulation contributes to endothelial dysfunction and stroke susceptibility.

## 5 Conclusion

This nationwide cross-sectional analysis revealed a significant link between UHR and increased overall self-reported stroke prevalence among US adults. These findings offer new insights into metabolic biomarkers in stroke prevalence stratification and support the potential utility of UHR for cross-sectional population-level discrimination rather than prognostic prediction or causal inference. The exploratory hospital-based imaging-confirmed hemorrhagic stroke analysis provided directional support in a clinically distinct phenotype, but these results should be interpreted as hypothesis-generating. Future longitudinal and mechanistic studies are needed to better understand the metabolic contribution of UHR to cerebrovascular vulnerability.

## Data Availability

The raw data supporting the conclusions of this article will be made available by the authors, without undue reservation.
