# Supplementary figures and images for: Serum uric acid-to-HDL cholesterol ratio and stroke prevalence: NHANES 1999–2018 with external support from an imaging-confirmed hemorrhagic stroke dataset
Source: Front Neurol. 2026 Jun 26;17:1798258. doi: 10.3389/fneur.2026.1798258 (PMC13349932; doi:10.3389/fneur.2026.1798258)

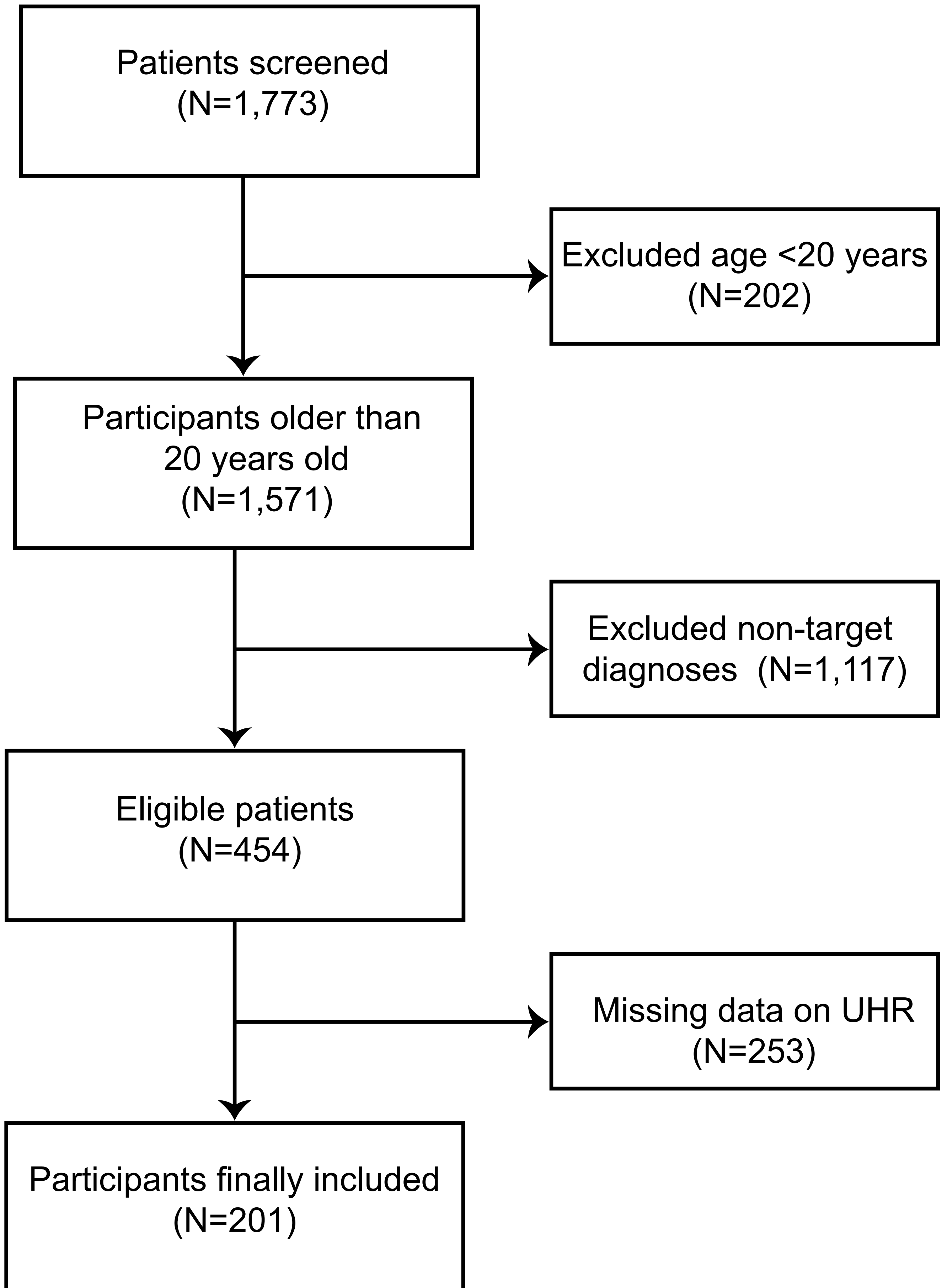

Supplement: Supplementary file 1 [file Data_Sheet_1.PDF]

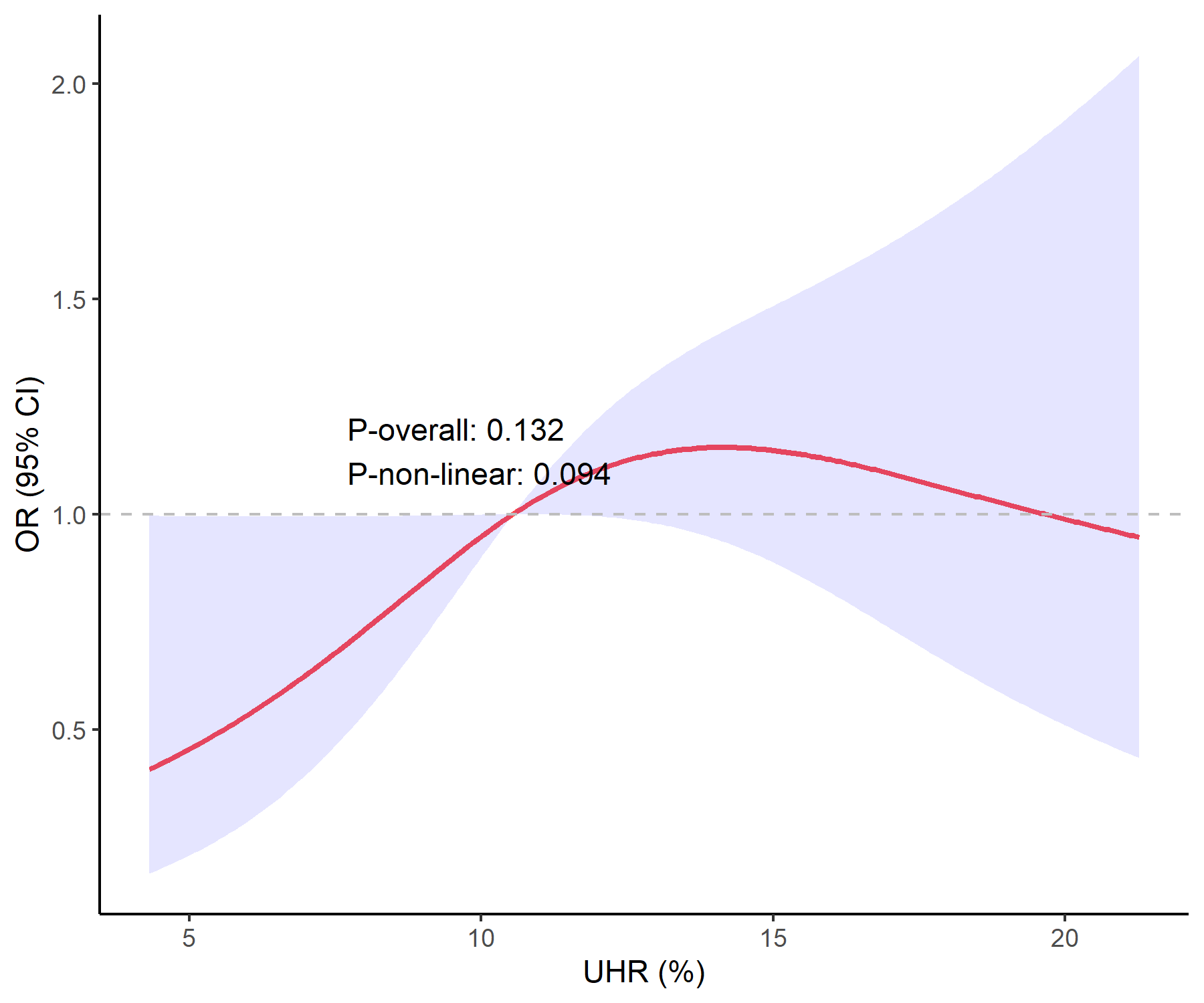

Supplement: Supplementary file 2 [file Image_1.PNG]
