# Supplementary material for: Serum uric acid-to-HDL cholesterol ratio and stroke prevalence: NHANES 1999–2018 with external support from an imaging-confirmed hemorrhagic stroke dataset
Source: Front Neurol. 2026 Jun 26;17:1798258. doi: 10.3389/fneur.2026.1798258 (PMC13349932; doi:10.3389/fneur.2026.1798258)
Supplement: Supplementary file 3 [file Table_1.DOCX]

**Table S1. Baseline characteristics of the external validation cohort stratified by UHR quartiles**

| **Characteristics** | **Q1 (≤ 7.93)**  **n (%)** | **Q2 (7.93–10.54)**  **n (%)** | **Q3 (10.54–14.22)**  **n (%)** | **Q4 (≥14.22)**  **n (%)** | **P value** |
| --- | --- | --- | --- | --- | --- |
| **Age (years)** | 56.88 ± 11.28 | 56.12 ± 12.30 | 55.30 ± 13.08 | 54.32 ± 13.23 | 0.759 |
| **Gender** |  |  |  |  | <0.001 |
| Male | 9 (17.6%) | 22 (44.0%) | 24 (48.0%) | 34 (68.0%) |  |
| Female | 42 (82.4%) | 28 (56.0%) | 26 (52.0%) | 16 (32.0%) |  |
| **Drinking status** |  |  |  |  | <0.001 |
| No | 48 (94.1%) | 38 (76.0%) | 37 (74.0%) | 26 (52.0%) |  |
| Yes | 3 (5.9%) | 12 (24.0%) | 13 (26.0%) | 24 (48.0%) |  |
| **Smoking status** |  |  |  |  | 0.002 |
| No | 47 (92.2%) | 40 (80.0%) | 37 (74.0%) | 30 (60.0%) |  |
| Yes | 4 (7.8%) | 10 (20.0%) | 13 (26.0%) | 20 (40.0%) |  |
| **Hypertension** |  |  |  |  | 0.762 |
| No | 30 (58.8%) | 28 (56.0%) | 32 (64.0%) | 27 (54.0%) |  |
| Yes | 21 (41.2%) | 22 (44.0%) | 18 (36.0%) | 23 (46.0%) |  |
| **Diabetes** |  |  |  |  | 0.464 |
| No | 44 (86.3%) | 45 (90.0%) | 47 (94.0%) | 47 (94.0%) |  |
| Yes | 7 (13.7%) | 5 (10.0%) | 3 (6.0%) | 3 (6.0%) |  |
| **Body mass index (kg/m2)** | 23.38 ± 3.28 | 23.87 ± 3.83 | 24.03 ± 2.75 | 25.35 ± 3.53 | 0.027 |
| **HDL-C(mmol/dL)** | 60.36 ± 13.10 | 54.21 ± 9.46 | 48.45 ± 10.31 | 39.59 ± 6.89 | <0.001 |
| **LDL-C(mmol/L)** | 2.61 ± 0.91 | 2.71 ± 0.80 | 2.70 ± 0.82 | 2.54 ± 1.08 | 0.764 |
| **SUA（mg/dL）** | 3.48 ± 0.87 | 4.93 ± 0.86 | 5.96 ± 1.21 | 7.22 ± 1.58 | <0.001 |
| **Creatinine（umol/L）** | 57.86 ± 12.34 | 67.90 ± 23.89 | 79.42 ± 37.16 | 80.66 ± 23.76 | <0.001 |

Note: Data are mean ± SD for continuous variables and n (column %) for categorical variables. Percentages sum to 100% within columns unless stated. Abbreviations: HDL-C and LDL-C, high- and low-density lipoprotein cholesterol; SUA, serum uric acid.
