# Supplementary material for: Serum uric acid-to-HDL cholesterol ratio and stroke prevalence: NHANES 1999–2018 with external support from an imaging-confirmed hemorrhagic stroke dataset
Source: Front Neurol. 2026 Jun 26;17:1798258. doi: 10.3389/fneur.2026.1798258 (PMC13349932; doi:10.3389/fneur.2026.1798258)
Supplement: Supplementary file 4 [file Table_2.DOCX]

**Table S2. Baseline characteristics of the external validation cohort according to hemorrhage stroke.**

| **Characteristics** | **Total(N=201)** | **Non-Stroke(N=130)** | **Hemorrhage stroke (N=71)** | ***P* value** |
| --- | --- | --- | --- | --- |
| **Age (years)** | 55.66 ± 12.43 | 55.08 ± 12.60 | 56.73 ± 12.13 | 0.621 |
| **Gender** |  |  |  | 0.752 |
| Male**,** n (%) | 89 (44.3%) | 56 (43.1%) | 33 (46.5%) |  |
| Female, n (%) | 112 (55.7%) | 74 (56.9%) | 38 (53.5%) |  |
| **Drinking status** |  |  |  | 0.770 |
| No | 149 (74.1%) | 95 (73.1%) | 54 (76.1%) |  |
| Yes | 52 (25.9%) | 35 (26.9%) | 17 (23.9%) |  |
| **smoking status** |  |  |  | 0.754 |
| No, n (%) | 154 (76.6%) | 101 (77.7%) | 53 (74.6%) |  |
| Yes, n (%) | 47 (23.4%) | 29 (22.3%) | 18 (25.4%) |  |
| **Hypertension** |  |  |  | <0.001 |
| No, n (%) | 117 (58.2%) | 88 (67.7%) | 29 (40.8%) |  |
| Yes, n (%) | 84 (41.8%) | 42 (32.3%) | 42 (59.2%) |  |
| **Diabetes** |  |  |  | 0.555 |
| No, n (%) | 183 (91.0%) | 120 (92.3%) | 63 (88.7%) |  |
| Yes, n (%) | 18 (9.0%) | 10 (7.7%) | 8 (11.3%) |  |
| **Body mass index (kg/m2)** | 24.15 ± 3.42 | 24.02 ± 3.29 | 24.39 ± 3.66 | 0.400 |
| **HDL-C(mg/dL)** | 50.70 ± 12.71 | 51.75 ± 13.07 | 48.77 ± 11.86 | 0.089 |
| **LDL-C(mmol/L)** | 2.64 ± 0.90 | 2.66 ± 0.92 | 2.61 ± 0.89 | 0.811 |
| **SUA（mg/dL）** | 5.39 ± 1.80 | 5.27 ± 1.77 | 5.60 ± 1.86 | 0.263 |
| **Creatinine（umol/L）** | 71.39 ± 27.24 µmol/L | 69.42 ± 21.40 µmol/L | 75.01 ± 35.42 µmol/L | 0.322 |
| **UHR** | 11.48 ± 5.30 | 11.14 ± 5.50 | 12.08 ± 4.90 | 0.090 |

Note: Data are mean ± SD for continuous variables and n (column %) for categorical variables. Percentages sum to 100% within columns unless stated. Abbreviations: HDL-C and LDL-C, high- and low-density lipoprotein cholesterol; SUA, serum uric acid; UHR = (SUA [mg/dL] / HDL-C [mg/dL]) × 100.
