# Supplementary material for: Serum uric acid-to-HDL cholesterol ratio and stroke prevalence: NHANES 1999–2018 with external support from an imaging-confirmed hemorrhagic stroke dataset
Source: Front Neurol. 2026 Jun 26;17:1798258. doi: 10.3389/fneur.2026.1798258 (PMC13349932; doi:10.3389/fneur.2026.1798258)
Supplement: Supplementary file 5 [file Table_3.DOCX]

**Table S3. The Association of UHR with hemorrhage stroke in the external validation cohort.**

| **Exposure** | **OR (95%CI), p-value** | | |
| --- | --- | --- | --- |
|  | **Model 1^a^** | **Model 2^b^** | **Model 3^c^** |
| **UHR** | 1.03 (0.98-1.09), 0.232 | 1.04 (0.98-1.10), 0.220 | 0.99 (0.91-1.08), 0.848 |
| **UHR quartile** |  |  |  |
| **Q1** | 1 | 1 | 1 |
| **Q2** | 1.51 (0.64-3.56), 0.351 | 1.54 (0.64-3.70), 0.336 | 1.51 (0.59-3.87), 0.391 |
| **Q3** | 2.12 (0.91-4.92), 0.082 | 2.19 (0.92-5.22), 0.076 | 2.18 (0.78-6.06), 0.136 |
| **Q4** | 1.95 (0.84-4.54), 0.122 | 2.06 (0.83-5.10), 0.118 | 1.67 (0.50-5.59), 0.402 |
| **AIC** | 263.64 | 266.50 | 264.40 |
| **BIC** | 270.25 | 279.72 | 303.98 |
| **Pseudo R²** | 0.005 | 0.010 | 0.076 |
| **AUC** | 0.572 | 0.542 | 0.693 |

^a^No-adjusted model: unadjusted.

^b^Minimally adjusted model: adjusted for age and sex.

^c^Fully adjusted model: further adjusted for hypertension, diabetes mellitus, body mass index, smoking status, drinking status, LDL-C, and serum creatinine.
